# Association between daytime nap duration and risks of frailty: Findings from the China Health and Retirement Longitudinal Study

**DOI:** 10.3389/fpubh.2022.1098609

**Published:** 2023-01-27

**Authors:** Yan Zhang, Lixing Zhou, Meiling Ge, Xiufang Lin, Birong Dong

**Affiliations:** ^1^Center of Gerontology and Geriatrics, West China Hospital, Sichuan University, Chengdu, Sichuan, China; ^2^National Clinical Research Center for Geriatrics, West China Hospital, Sichuan University, Chengdu, Sichuan, China

**Keywords:** nap, sleep duration, frailty, older adults, CHARLS

## Abstract

**Introduction:**

Night sleep duration and total sleep duration are associated with frailty. However, the association between daytime nap duration and the risks of frailty has not been explored thoroughly.

**Methods:**

This study used data from the China Health and Retirement Longitudinal Study (CHARLS). Participants aged 60 years and older at baseline were included in this study. Individuals with daytime nap duration were categorized into four groups: no napping, short napping (< 30 min), moderate napping (30–89 min), and extended napping (≥90 min). Frailty was assessed using a modified Physical Frailty Phenotype (PFP) scale. Non-frail participants at baseline were followed up for 4 years. The association between nap duration and risks of frailty at baseline and incident frailty was evaluated by logistic regression and discrete-time Cox regression analyses, respectively.

**Results:**

In total, 5,126 participants were included in this study. For individuals with night sleep duration of ≥9 h, short nappers showed higher odds [odds ratio (OR) = 4.08, 95% confidence interval (CI): 1.30–12.78] for frailty compared with non-habitual nappers at baseline, while moderate nappers were less likely to be frail (OR = 0.18, 95% CI: 0.04–0.73). In the follow-up study, short nappers showed higher risks for frailty compared with participants of the no napping group with night sleep duration of < 6 h [hazard ratio (HR) = 1.91, 95% CI: 1.07–3.43] or 6–9 h (HR = 1.97, 95% CI: 1.18–3.30). Compared with short nappers, older adults with extended napping (HR = 0.41, 95% CI: 0.22–0.77) showed lower risks for frailty in those with night sleep duration of 6–9 h. For individuals with night sleep duration of ≥9 h, moderate napping (HR = 0.20, 95% CI: 0.05–0.77) decreased the risks for frailty compared with short napping.

**Conclusion:**

Among older adults with night sleep duration of < 9 h, short nappers posed higher risks for frailty compared with non-habitual nappers. Extended naps for those with a night sleep duration of 6–9 h or moderate naps for those with night sleep duration of ≥9 h could lower the risk of frailty compared with short naps. Future studies on the timing, purpose, frequency, and quality of daytime napping and objectively measured nap duration are needed to explore the association between daytime napping and risks of frailty.

## Introduction

Frailty is an age-related clinical syndrome that is characterized by an increased vulnerability to stressors caused by a cumulative decline in multiple physiologic systems (1, 2). Frailty could increase the risk of adverse outcomes, such as disability, hospitalization, falls, and death, which would be a threat to the quality of life and impose a heavy economic burden on medical treatment and caregiving (3). However, frailty is not an irreversible condition. It has been shown that interventions targeted at risk factors for frailty may be effective strategies for frailty prevention and recovery (4, 5).

Sleep condition, especially sleep duration, is one of the risk factors which has been reported to be associated with frailty. According to two recent systematic reviews, both short and long sleep duration were associated with frailty (6, 7). However, since most of the studies included were cross-sectional, causal relationships between sleep duration and frailty could not be inferred. A longitudinal study showed that both short and long sleep duration were associated with incident frailty in Mexico (8), whereas Chen et al. reported that only long sleep duration was associated with increased risks of frailty among older adults in China (9). Another study found that short sleep duration was not associated with frailty at follow-up investigations (10). The aforementioned longitudinal studies on the association of sleep duration and risks of frailty barely investigated the effects of daytime nap duration or calculated only the total sleep duration per day and did not treat daytime nap duration as a primary independent variable.

Napping, an important part of sleep behavior, is very much prevalent among older adults (11–13). According to studies based on a nationally representative survey, more than half of the older adults were habitual nappers in China (14–16). Daytime napping, as a modifiable lifestyle factor impacting health, has been reported to increase or decrease the risks of adverse outcomes, such as cognitive decline, hypertension, diabetes, metabolic syndrome, stroke, and mortality (12, 17–21). However, a few studies focused on napping and the risks of frailty. A cross-sectional study in China that combined frailty and cognitive impairment found that long nap duration was associated with higher odds of cognitive frailty and physical frailty among older adults in nursing homes (22). Another cross-sectional study showed that long nap duration was associated with a lower likelihood of successful aging among community-dwelling older adults in China (23). Owing to the limited number and cross-sectional design of previous studies, knowledge gap in the association between napping and risks of frailty are still prevalent. Therefore, this study aimed to identify the relationship between daytime napping and the risks of frailty using data from the China Health and Retirement Longitudinal Study (CHARLS), a large sample size longitudinal study from China.

## Methods

### Study population

**Figure 1:**
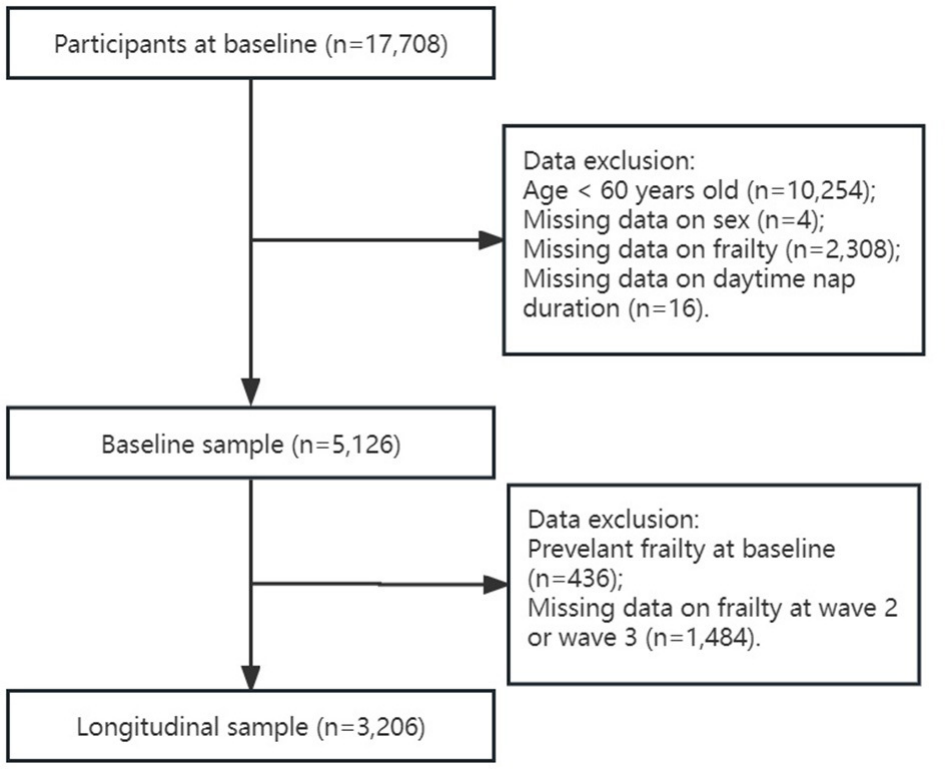
Flow chart of sample selection.

Data were obtained from the China Health and Retirement Longitudinal Study (CHARLS), a nationally representative longitudinal survey on community-dwelling adults aged 45 years and older from 28 provinces in China. The CHARLS was started in 2011 and included 17,708 participants at baseline, with follow-up surveys conducted every 2 years thereafter (2013, 2015, and 2018). Details of the CHARLS have been described previously (24). All participants provided informed consent. Ethical approval for data collection of all the CHARLS waves was obtained from the Institutional Review Board at Peking University (IRB00001052–11015). The present study used only data from baseline, wave 2 (2013), and wave 3 (2015), because wave 4 (2018) did not contain sufficient data on physical frailty phenotype. To focus better on older adults suffering from frailty, individuals aged 60 years and older were included in this study. In baseline data analyses, individuals with missing data on daytime napping time or frailty were excluded from this study. Participants with frailty at baseline in predicting the risk of developing frailty in the following surveys at wave 2 and wave 3 were excluded further from this study (see flowchart in Figure 1).

### Measurements

#### Frailty

Frailty was measured using the modified Physical Frailty Phenotype (PFP) scale (25, 26) that consists of five criteria: weakness, slowness, exhaustion, shrinking, and inactivity. Individuals meeting three or more criteria were considered frail; otherwise, they were deemed non-frail.

#### Weakness

Weakness was defined using the maximum of the two-timed hand grip strength test of either hand, as being ≤ 20th percentile of the population within the four categories adjusted for sex and body mass index (BMI).

#### Slowness

Slowness was defined using the average gait speed of the two-timed walking tests over 2.5 m, as being ≤ 20th percentile of the population within the four categories classified by sex and sex-specific median height.

#### Exhaustion

Participants were asked if they could not get going or felt everything they did was an effort during the last week. Individuals who answered “Occasionally or a moderate amount of time (3–4 days)” or “Most or all the time (5–7 days)” to either of these two conditions were classified as exhausted.

#### Shrinking

Shrinking was defined as the self-reported weight loss of ≥5 kg in the previous year at baseline or a loss of ≥10% weight compared to the previous wave (in wave 2 and wave 3) or having a BMI of 18.5 kg/m^2^ or less (27).

#### Inactivity

Participants were classified as being inactive if they answered no to the question if they walked for at least 10 min continuously in the course of a usual week.

#### Daytime nap duration

Daytime nap duration was assessed from the response to the following question: “During the past month, how long did you take a nap after lunch?” In accordance with previous studies (16, 28), individuals were categorized into four groups: no napping, 0 min per day; short napping, < 30 min per day (not including 0 min); moderate napping, 30–89 min per day; and extended napping, ≥90 min per day.

#### Covariates

Covariates consisted of demographic variables and health and function variables. Demographic variables included age, sex, marital status (married, widowed, or others), current residence (urban or rural), and education level (no formal education or illiterate, did not finish elementary school, elementary school, middle school, or high school or above). Health and function variables included smoking (non-smoker, ex-smoker, or current smoker), drinking (never, drinks occasionally, or drinks frequently), number of chronic conditions, cognitive function, depression (no or yes), and night sleep duration. Drinking occasionally was defined as drinking less than one time a month in the last year. People drinking more than one time a month in the last year were categorized as drinking frequently. The number of chronic conditions was calculated by the total number of self-reported histories of hypertension, diabetes, cancer of the malignant tumor type, chronic lung diseases, liver disease, heart problems, stroke, kidney disease, stomach or other digestive diseases, and arthritis or rheumatism. According to the total number of chronic conditions, participants were divided into three groups (0, 1, and >1). Cognitive function was assessed by the Telephone Interview of Cognitive Status scale (TICS-10), episodic memory, and visuospatial abilities. The total score ranged from 0 to 21 and a higher score represented better cognitive function (29). Depression symptoms were assessed using the modified 10-item Center for Epidemiologic Studies Depression scale (CESD-10) (30), and individuals with a total score of 12 or more were categorized as having depression (31). Night sleep duration was assessed from the response to the following question: “During the past month, how many hours of actual sleep did you get at night?” Based on previous literature (32), participants were classified into three groups (< 6, 6–9, or ≥9 h per day) according to their average sleep duration per night during the past month.

### Statistical analyses

Descriptive analyses were presented as the mean ± standard deviation for normally distributed variables, the median (quartile) for abnormally distributed variables, and number (percentage) for categorical variables. Baseline characteristics of frailty status and covariates were grouped by nap duration and compared using *t*-tests, Wilcoxon's rank-sum test, and Pearson's chi-square test for normally distributed, abnormally distributed, and categorical variables, respectively. Logistic regression analysis was conducted to determine the association between nap duration and the risks of frailty at baseline, and discrete-time Cox regression analysis was used to examine the association between daytime napg duration and incident frailty in the follow-up studies. Subgroup analyses stratified by night sleep duration were performed in the aforementioned analyses. Multivariate analyses included three models: Model 1 was unadjusted. Model 2 was adjusted for age and sex; and Model 3 was adjusted for age, sex, marital status, current residence, education, smoking, drinking, the number of chronic conditions, cognitive function, depression, and night sleep duration.

Sensitivity analyses were performed. Mortality and/or incident frailty were set as composite outcomes to examine the competing effect of mortality for frailty.

Stata 15.1 (Stata Corp, College Station, TX, USA) was used for data analyses. A two-sided value of *p* < 0.05 was considered statistically significant.

## Results

### Sample characteristics

Table 1 shows the characteristics of the study population. A total of 5,126 participants were included in this study, of whom 2,257 (44.0%) individuals were non-habitual nappers, 480 (9.4%) older adults took naps < 30 min per day, 1,607 (31.3%) participants took naps 30–89 min per day, and 782 (15.3%) extended nappers took naps ≥90 min per day during the past month at baseline. The average age of the participants was 67.7 ± 6.4 years, with 48.7% of participants being women. The prevalence of frailty showed no difference between the four nap duration groups. The non-habitual napping group showed a higher percentage of women (no napping: 56.7%, short napping: 45.8%, moderate napping: 43.7%, and extended napping: 37.5%), a higher percentage of rural residents (no napping: 82.8%, short napping: 73.8%, moderate napping: 73.0%, and extended napping: 82.1%), a higher prevalence of depression (no napping: 35.4%, short napping: 31.8%, moderate napping: 28.1%, and extended napping: 25.1%), and a lower cognition score (no napping: 9.5, short napping: 11.0, moderate napping: 11.0 and extended napping: 10.5). Extended napping group showed longer night sleep duration than other groups (no napping: 6.0, short napping: 6.0, moderate napping: 6.0, and extended napping: 7.0).

**Table 1:** Baseline characteristics of study sample grouped by daytime nap duration, the China Health and Retirement Longitudinal Study (*n* = 5,126).

**Characteristics**		**Overall**	**Daytime nap duration**				***P-value***
		**(*****n*** = **5,126)**	**No napping** ***n*** = **2,257 (44.0%)**	**Short napping** ***n*** = **480 (9.4%)**	**Moderate napping** ***n*** = **1,607 (31.3%)**	**Extended napping** ***n*** = **782 (15.3%)**	
Age, mean ± SD		67.7 ± 6.4	67.5 ± 6.3	67.5 ± 6.3	67.9 ± 6.5	68.0 ± 6.5	0.16
Sex, *n* (%)	Male	2,630 (51.3)	977 (43.3)	260 (54.2)	904 (56.3)	489 (62.5)	< 0.001
	Female	2,496 (48.7)	1,280 (56.7)	220 (45.8)	703 (43.7)	293 (37.5)	
Marital status, *n* (%)	Married	4,059 (79.2)	1,733 (76.8)	381 (79.4)	1,308 (81.4)	637 (81.5)	0.013
	Widowed	961 (18.7)	471 (20.9)	89 (18.5)	267 (16.6)	134 (17.1)	
	Others	106 (2.1)	53 (2.3)	10 (2.1)	32 (2.0)	11 (1.4)	
Current residence, *n* (%)	Urban	1,087 (21.2)	388 (17.2)	126 (26.3)	433 (27.0)	140 (17.9)	< 0.001
	Rural	4,035 (78.8)	1,868 (82.8)	354 (73.8)	1,171 (73.0)	642 (82.1)	
Education, *n* (%)	No formal education or illiterate	1,838 (35.9)	945 (41.9)	141 (29.4)	499 (31.1)	253 (32.4)	< 0.001
	Did not finish elementary school	1,018 (19.9)	442 (19.6)	75 (15.6)	340 (21.2)	161 (20.6)	
	Elementary school	1,344 (26.2)	556 (24.6)	145 (30.2)	419 (26.1)	224 (28.6)	
	Middle school	617 (12.0)	218 (9.7)	89 (18.5)	214 (13.3)	96 (12.3)	
	High school or above	308 (6.0)	95 (4.2)	30 (6.3)	135 (8.4)	48 (6.1)	
Smoking, *n* (%)	Non-smoker	2,897 (56.5)	1,370 (60.7)	274 (57.1)	881 (54.8)	372 (47.6)	< 0.001
	Ex-smoker	624 (12.2)	237 (10.5)	67 (14.0)	217 (13.5)	103 (13.2)	
	Current smoker	1,605 (31.3)	650 (28.8)	139 (29.0)	509 (31.7)	307 (39.3)	
Drinking, *n* (%)	Never	3,535 (69.0)	1,656 (73.4)	338 (70.4)	1,060 (66.0)	481 (61.5)	< 0.001
	Drink occasionally	351 (6.8)	119 (5.3)	36 (7.5)	129 (8.0)	67 (8.6)	
	Drink frequently	1,240 (24.2)	482 (21.4)	106 (22.1)	418 (26.0)	234 (29.9)	
Number of chronic conditions, *n* (%)	0	1,387 (27.6)	665 (30.1)	125 (26.6)	380 (24.1)	217 (28.1)	< 0.001
	1	1,596 (31.8)	705 (31.9)	142 (30.2)	488 (31.0)	261 (33.8)	
	>1	2,043 (40.6)	839 (38.0)	203 (43.2)	707 (44.9)	294 (38.1)	
Cognition score, median (IQR)		10.5 (6.5, 13.5)	9.5 (5.5, 13.0)	11.0 (7.0, 13.5)	11.0 (7.0, 13.5)	10.5 (6.5, 13.5)	< 0.001
Depression, *n* (%)	No	3,361 (68.8)	1,383 (64.6)	311 (68.2)	1,106 (71.9)	561 (74.9)	< 0.001
	Yes	1,525 (31.2)	759 (35.4)	145 (31.8)	433 (28.1)	188 (25.1)	
Night sleep duration, *n* (%)	< 6 h	1,743 (34.3)	912 (40.8)	149 (31.2)	496 (31.1)	186 (23.9)	< 0.001
	6–9 h	2,931 (57.6)	1,153 (51.6)	286 (60.0)	997 (62.4)	495 (63.5)	
	≥9 h	412 (8.1)	168 (7.5)	42 (8.8)	104 (6.5)	98 (12.6)	
Night sleep duration, median (IQR)		6.0 (5.0, 8.0)	6.0 (4.0, 8.0)	6.0 (5.0, 8.0)	6.0 (5.0, 8.0)	7.0 (6.0, 8.0)	< 0.001
Frailty, *n* (%)	Non-frail	4,690 (91.5)	2,054 (91.0)	439 (91.5)	1,482 (92.2)	715 (91.4)	0.62
	Frail	436 (8.5)	203 (9.0)	41 (8.5)	125 (7.8)	67 (8.6)	

SD, standard deviation; IQR, interquartile range.

### Association between daytime nap duration and the risks of frailty at baseline

No association was found between daytime nap duration and the risks of frailty at baseline in the unadjusted model and adjusted model (Table 2). However, in the subgroup analyses stratified by night sleep duration, this study found that, among older adults with night sleep duration of ≥9 h, short nappers showed higher odds (OR = 4.08, 95% CI: 1.30–12.78) for frailty in the fully adjusted model compared with the no napping group, while moderate nappers showed lower odds for frailty in Model 2 (OR = 0.28, 95% CI: 0.09–0.88) and Model 3 (OR = 0.18, 95% CI: 0.04–0.73).

**Table 2:** Association of daytime nap duration and frailty at baseline from the China Health and Retirement Longitudinal Study (*n* = 5,126).

	**No napping**	**Short napping**		**Moderate napping**		**Extended napping**	
		**OR**	**95%CI**	**OR**	**95%CI**	**OR**	**95%CI**
Model 1	Reference	0.94	(0.67, 1.34)	0.85	(0.68, 1.08)	0.95	(0.71, 1.27)
Model 2	Reference	0.96	(0.67, 1.37)	0.82	(0.65, 1.04)	0.91	(0.68, 1.23)
Model 3	Reference	1.08	(0.73, 1.60)	0.97	(0.74, 1.26)	1.09	(0.78, 1.52)
**Subgroup analyses** **Night sleep duration**<**6 h**							
Model 1	Reference	1.03	(0.61, 1.76)	1.13	(0.82, 1.58)	0.91	(0.55, 1.50)
Model 2	Reference	1.03	(0.59, 1.77)	1.07	(0.76, 1.51)	0.87	(0.52, 1.47)
Model 3	Reference	0.96	(0.53, 1.73)	1.18	(0.82, 1.71)	1.06	(0.59, 1.89)
**Night sleep duration 6–9 h**							
Model 1	Reference	0.74	(0.41, 1.33)	0.82	(0.57, 1.18)	1.13	(0.75, 1.70)
Model 2	Reference	0.75	(0.41, 1.36)	0.79	(0.55, 1.15)	1.08	(0.71, 1.65)
Model 3	Reference	0.82	(0.43, 1.57)	0.97	(0.65, 1.47)	1.19	(0.75, 1.90)
**Night sleep duration** ≥**9 h**							
Model 1	Reference	2.27	(0.94, 5.50)	0.33	(0.11, 1.01)	0.95	(0.42, 2.14)
Model 2	Reference	2.10	(0.84, 5.23)	0.28^*^	(0.09, 0.88)	0.80	(0.34, 1.86)
Model 3	Reference	4.08^*^	(1.30, 12.78)	0.18^*^	(0.04, 0.73)	0.61	(0.21, 1.76)

OR: odds ratio; 95%CI: confidence interval;

* *p* < 0.05.

Model 1: unadjusted model; Model 2: adjusted for age and sex; Model 3: adjusted for age, sex marital status, current residence, education level, smoking, drinking, number of chronic conditions, cognitive function, depression and night sleep duration (in the unstratified analyses).

### Association between daytime nap duration and incident frailty in the follow-up surveys

The association between daytime nap duration and incident frailty in the follow-up surveys is shown in Table 3. Compared with the no napping group, older adults with a nap duration of < 30 min showed a higher risk for incident frailty in all three models (Model 1: HR = 1.50, 95% CI: 1.06–2.13; Model 2: HR = 1.62, 95% CI: 1.14–2.29; and Model 3: HR = 2.01, 95% CI: 1.40–2.88). In subgroup analyses stratified by night sleep duration, a greater risk of incident frailty was found in the adjusted models for people with night sleep duration of < 6 h (Model 3: HR = 1.91, 95% CI: 1.07–3.43) and 6–9 h (Model 2: HR = 1.69, 95% CI: 1.02–2.80; and Model 3: HR = 1.97, 95% CI: 1.18–3.30), respectively. However, no association was found for participants with night sleep duration of ≥9 h.

**Table 3:** Association of daytime nap duration and incident frailty in the follow-up surveys (wave2–wave3) from the China Health and Retirement Longitudinal Study (*n* = 3,206).

	**No napping**	**Short napping**		**Moderate napping**		**Extended napping**	
		**HR**	**95%CI**	**HR**	**95%CI**	**HR**	**95%CI**
Model 1	Reference	1.50^*^	(1.06, 2.13)	1.02	(0.79, 1.33)	0.87	(0.62, 1.24)
Model 2	Reference	1.62^**^	(1.14, 2.29)	1.04	(0.80, 1.36)	0.87	(0.61, 1.24)
Model 3	Reference	2.01^***^	(1.40, 2.88)	1.16	(0.87, 1.54)	0.96	(0.66, 1.40)
**Subgroup analyses** **Night sleep duration**<**6 h**							
Model 1	Reference	1.59	(0.90, 2.79)	1.08	(0.71, 1.64)	1.17	(0.67, 2.06)
Model 2	Reference	1.54	(0.88, 2.72)	1.09	(0.71, 1.65)	1.08	(0.61, 1.91)
Model 3	Reference	1.91^*^	(1.07, 3.43)	1.28	(0.82, 2.00)	1.25	(0.69, 2.28)
**Night sleep duration 6–9 h**							
Model 1	Reference	1.52	(0.92, 2.51)	1.12	(0.77, 1.64)	0.81	(0.48, 1.36)
Model 2	Reference	1.69^*^	(1.02, 2.80)	1.17	(0.80, 1.72)	0.83	(0.49, 1.40)
Model 3	Reference	1.97^**^	(1.18, 3.30)	1.28	(0.85, 1.90)	0.81	(0.47, 1.39)
**Night sleep duration** ≥**9 h**							
Model 1	Reference	2.12	(0.81, 5.52)	0.69	(0.28, 1.71)	0.80	(0.32, 1.97)
Model 2	Reference	2.04	(0.78, 5.35)	0.61	(0.24, 1.53)	0.78	(0.31, 1.95)
Model 3	Reference	2.91	(0.87, 9.75)	0.57	(0.19, 1.73)	0.78	(0.26, 2.28)

HR: hazards ratio; 95%CI: 95% confidence interval;

* p < 0.05,

** p < 0.01,

*** p < 0.001.

Model 1: unadjusted model; Model 2: adjusted for age and sex; Model 3: adjusted for age, sex marital status, current residence, education level, smoking, drinking, number of chronic conditions, cognitive function, depression and night sleep duration (in the unstratified analyses).

The present study examined further the association between nap duration and incident frailty when setting short napping as the reference group to find out whether moderate-to-long napping could lower the risks of frailty compared with short napping among habitual nappers (Supplementary Table S1). The results showed that, compared with the short napping group, individuals with no napping or moderate-to-long nap duration all had decreased risks for frailty. In subgroup analyses, for individuals with night sleep duration of 6–9 h, both no napping (HR = 0.51, 95% CI: 0.30–0.85) and extended napping (HR = 0.41, 95% CI: 0.22–0.77) showed lower risks of frailty compared with short napping. For individuals with night sleep duration of ≥9 h, moderate napping (HR = 0.20, 95% CI: 0.05–0.77) showed lower risks of frailty compared with short napping.

### Sensitivity analyses

The results of sensitivity analyses were similar when exploring the association between daytime napping duration and incident mortality and/or frailty as a composite outcome (Supplementary Table S2).

## Discussion

Although several studies showed that night sleep duration and total sleep duration were associated with frailty, daytime nap duration has not been investigated or considered as a primary independent factor in these studies. The relationship between daytime nap duration and the risks of frailty lacks clarity. The present study, therefore, aimed to explore the association between daytime nap duration and risks of frailty in a longitudinal study.

Napping is very common among older adults in China. They take naps for different reasons, including compensation for insufficient night sleep, beliefs in the beneficial impact of napping on health, low energy level, or feeling bored (33). According to the research mentioned in this study, over half of the older adults were habitual nappers, among whom more than half of them took naps with moderate duration (30–89 min).

In the baseline analyses, the present study found that, among participants with a night sleep duration of ≥9 h, those with short nap duration showed higher odds to be frail compared with those of the no napping group, while those with moderate nap duration showed the opposite result. In the longitudinal analyses of nap duration and incident frailty, older adults with short nap duration showed higher risks of frailty compared with non-habitual nappers. After stratification by night sleep duration, the association between short napping and higher risks of frailty was still significant for those with a night sleep duration of < 9 h. The results of the present study were inconsistent with those of a previous cross-sectional study regarding nap duration and cognitive frailty, which indicated that short nap duration was associated with lower odds for physical prefrail, frailty, and cognitive frailty, while longer nap duration increased the odds for all three conditions (22). The discrepancy could be attributed to the different study populations. The former study was conducted among older adults living in nursing homes, while the present study was conducted among community-dwelling residents. In addition, the cross-sectional study design and different frailty assessment tools (i.e., the Fatigue, Resistance, Aerobic capacity, Illnesses, and Loss of weight (FRAIL) scale) used in the previous study could be the reasons for the different results. Nevertheless, a study focusing on the effects of short naps after sleep deprivation on physical performance found that a 20-min post-lunch nap after sleep deprivation is not sufficient for the recovery from most of the physical performance and subjective fatigue among soccer players. However, the aforementioned research was conducted among athletes, while a limited number of studies focused on napping and the incidence of frailty among older adults. The mechanisms of negative effects of short nap duration on incident frailty need to be explored further.

The present study also showed that, for individuals with a night sleep duration of 6–9 h, naps >90 min could lower the risks for incident frailty compared with short naps. For older adults with a night sleep duration ≥9 h, no association was found between napping and the risks of frailty when compared with no napping; however, naps with moderate duration could decrease the risks for frailty compared with short naps. A previous study focused on daytime napping and successful aging, which was defined as the coexistence of low probability of disease, no disease-related disability, high physical functioning, and active engagement with life for older adults, suggesting that those with daytime nap duration of >60 min per day had lower odds for successful aging compared with non-habitual nappers among older adults with night sleep duration of ≥8 h per night (23). Although successful aging and physical frailty are both age-related conditions and can both reflect the health status of older adults, the assessment tools of these two conditions contain different items and could result in different associations with daytime nap duration. Nevertheless, a systematic review reported that a longer nap, with a duration of 90 min suggested as the optimal, could result in an improvement in physical performance and decrease fatigue among athletes (34). The author speculated that a longer nap (i.e., 90 min) enables a complete sleep cycle with both non-rapid eye movement (NREM) and rapid eye movement (REM). NREM is beneficial for body restoration, which may result in higher performance (35). Therefore, compared with shorter naps, longer naps provide a sufficient time for NREM for body recovery, and individuals could wake up at the REM, which could reduce the severity of sleep inertia (36). In this vein, naps with longer duration may improve performance in components of frailty, such as slowness and exhaustion, compared with short naps. However, current studies on daytime nap duration and components of frailty were conducted among young people. Research on the effects of nap duration on the development of frailty or items of frailty among older adults is needed in future studies.

The present study has several strengths. First, to our knowledge, this is the first study to explore the longitudinal association between daytime nap duration and the risks of frailty among older adults in China. Second, the CHARLS used in the present study is a nationally representative study with a large sample size of community-dwelling older adults in China. Furthermore, frailty in this study was assessed by a well-validated tool (26).

This study has some limitations. First, night sleep duration and daytime nap duration were all self-reported. Thus, an underestimation of nap duration among older adults may be possible, which could inevitably cause recall bias and lead to misclassification (37, 38). Studies with an objectively measured night sleep duration and daytime nap duration are expected. Second, compared with people included in this study, those who were excluded due to missing data on nap duration or frail status were older, and a higher percentage of them belong to female sex, were urban residents, had depression, and showed lower cognition scores (Supplementary Table S3). This aspect could have introduced bias in this study. Third, data were used from the CHARLS did not contain information on the timing, purpose, frequency of napping, and quality of night sleep and daytime napping, which were reported to have implications for health status (39, 40). These factors should be considered while determining the effects of napping on risks of frailty in future studies. Additionally, the present study examined the relationship between nap duration and incident frailty in a 4-year study, followed up with three waves. A longer follow-up duration could be expected to find either a positive or a negative association between nap duration and risks of frailty. Owing to the transient improvement in physical performance and other health-related functions after naps mentioned in previous studies, the short-term association between napping and the components of frailty, such as slowness and exhaustion, could be explored through a quicker assessment after napping. Finally, in the follow-up surveys, mortality could have had a competing effect on frailty since people may die before the onset of frailty or die with frailty but have not been recorded because of the 2-year interval between the two waves. However, in the sensitivity analyses, the results were unchanged when mortality was combined with frailty as a composite outcome (Supplementary Table S2).

## Conclusion

Compared with non-habitual nappers, older adults with short napping were associated with higher risks of frailty for those with a night sleep duration of < 9 h. For participants with a night sleep duration of 6–9 h, long daytime napping could decrease risks of frailty compared with short napping. For those with a night sleep duration of ≥9 h, moderate napping could lower the risks of frailty compared with short napping. Therefore, for non-habitual nappers, there was no need to dissuade them from taking naps. For older adults with a daytime nap duration of < 30 min and a night sleep duration of ≥6 h per day, it would be better to extend their daytime nap duration to lower the risks of frailty. Given that napping habits are common and modifiable among older adults, it is of significance to change their napping habits to reduce the incidence of frailty and achieve healthy aging. Future studies should include an objectively measured nap duration, and information on the timing, frequency, purpose, and quality of napping is expected to provide a better understanding of the effect of napping on frailty and the underlying mechanisms behind them.

## Data availability statement

The datasets presented in this study can be found in online repositories. The names of the repository/repositories and accession number(s) can be found below: http://charls.pku.edu.cn.

## Ethics statement

The studies involving human participants were reviewed and approved by the Institutional Review Board at Peking University. The patients/participants provided their written informed consent to participate in this study.

## Author contributions

YZ and LZ contributed to the conception and design of this study. YZ and MG performed data analyses. YZ, LZ, MG, XL, and BD contributed to drafting and revising the article. All authors contributed to the article and approved the submitted version.
