# Supplementary material for: Association between daytime nap duration and risks of frailty: Findings from the China Health and Retirement Longitudinal Study
Source: Front Public Health. 2023 Jan 27;10:1098609. doi: 10.3389/fpubh.2022.1098609 (PMC9911424; doi:10.3389/fpubh.2022.1098609)
Supplement: Supplementary file 1 [file Table_1.docx]

**Table S1 Association of daytime nap duration and incident frailty from the China Health and Retirement Longitudinal Study (n=3 206)**

|  | No napping | | Short napping | Moderate napping | | Extended napping | |
| --- | --- | --- | --- | --- | --- | --- | --- |
|  | HR | 95%CI |  | HR | 95%CI | HR | 95%CI |
| Model 1 | 0.66* | (0.47,0.94) | Reference | 0.68* | (0.47,0.98) | 0.58* | (0.38,0.89) |
| Model 2 | 0.62** | (0.44,0.88) | Reference | 0.64* | (0.45,0.93) | 0.54** | (0.35,0.83) |
| Model 3 | 0.50*** | (0.35,0.71) | Reference | 0.58** | (0.40,0.84) | 0.48** | (0.31,0.75) |
| **Subgroup analyses** |  |  |  |  |  |  |  |
| **Night sleep duration<6h** |  |  |  |  |  |  |  |
| Model 1 | 0.63 | (0.36,1.10) | Reference | 0.68 | (0.37,1.24) | 0.74 | (0.36,1.51) |
| Model 2 | 0.65 | (0.37,1.14) | Reference | 0.70 | (0.38,1.29) | 0.70 | (0.34,1.43) |
| Model 3 | 0.52* | (0.29,0.94) | Reference | 0.67 | (0.35,1.27) | 0.66 | (0.31,1.38) |
| **Night sleep duration 6-9h** |  |  |  |  |  |  |  |
| Model 1 | 0.66 | (0.40,1.09) | Reference | 0.74 | (0.45,1.23) | 0.53* | (0.29,0.99) |
| Model 2 | 0.59* | (0.36,0.98) | Reference | 0.69 | (0.42,1.15) | 0.49* | (0.26,0.91) |
| Model 3 | 0.51** | (0.30,0.85) | Reference | 0.65 | (0.38,1.09) | 0.41** | (0.22,0.77) |
| **Night sleep duration ≥9h** |  |  |  |  |  |  |  |
| Model 1 | 0.47 | (0.18,1.23) | Reference | 0.32* | (0.11,0.97) | 0.38 | (0.13,1.12) |
| Model 2 | 0.49 | (0.19,1.28) | Reference | 0.30* | (0.10,0.90) | 0.38 | (0.13,1.14) |
| Model 3 | 0.34 | (0.10,1.15) | Reference | 0.20* | (0.05,0.77) | 0.27 | (0.07,1.02) |

HR: hazards ratio; 95%CI: 95% confidence interval; * p<0.05, ** p<0.01, *** p<0.001.

Model 1: unadjusted model; Model 2: adjusted for age and sex; Model 3: adjusted for age, sex marital status, current residence, education level, smoking, drinking, number of chronic conditions, cognitive function, depression and night sleep duration (in the unstratified analyses).
