# Supplementary material for: Association between daytime nap duration and risks of frailty: Findings from the China Health and Retirement Longitudinal Study
Source: Front Public Health. 2023 Jan 27;10:1098609. doi: 10.3389/fpubh.2022.1098609 (PMC9911424; doi:10.3389/fpubh.2022.1098609)
Supplement: Supplementary file 2 [file Table_2.docx]

**Table S2 Association of daytime nap duration and combined mortality and incident frailty from the China Health and Retirement Longitudinal Study (n=4 120)**

|  | No napping | Short napping | | Moderate napping | | Extended napping | |
| --- | --- | --- | --- | --- | --- | --- | --- |
|  |  | HR | 95%CI | HR | 95%CI | HR | 95%CI |
| Model 1 | Reference | 1.35* | (1.05,1.74) | 1.05 | (0.87,1.26) | 1.00 | (0.79,1.26) |
| Model 2 | Reference | 1.42** | (1.10,1.83) | 1.01 | (0.84,1.21) | 0.95 | (0.75,1.20) |
| Model 3 | Reference | 1.62*** | (1.24,2.11) | 1.09 | (0.89,1.32) | 1.01 | (0.79,1.29) |
| **Subgroup analyses** |  |  |  |  |  |  |  |
| **Night sleep duration<6h** |  |  |  |  |  |  |  |
| Model 1 | Reference | 1.57* | (1.04,2.37) | 1.15 | (0.85,1.55) | 1.21 | (0.80,1.81) |
| Model 2 | Reference | 1.52* | (1.00,2.30) | 1.12 | (0.83,1.52) | 1.12 | (0.74,1.68) |
| Model 3 | Reference | 1.64* | (1.05,2.57) | 1.24 | (0.90,1.71) | 1.27 | (0.82,1.96) |
| **Night sleep duration 6-9h** |  |  |  |  |  |  |  |
| Model 1 | Reference | 1.29 | (0.91,1.84) | 1.06 | (0.82,1.37) | 0.95 | (0.68,1.31) |
| Model 2 | Reference | 1.40 | (0.98,1.99) | 1.03 | (0.79,1.33) | 0.91 | (0.65,1.26) |
| Model 3 | Reference | 1.58* | (1.10,2.28) | 1.12 | (0.86,1.47) | 0.94 | (0.67,1.31) |
| **Night sleep duration ≥9h** |  |  |  |  |  |  |  |
| Model 1 | Reference | 1.54 | (0.73,3.27) | 0.87 | (0.48,1.57) | 0.94 | (0.51,1.72) |
| Model 2 | Reference | 1.41 | (0.66,3.01) | 0.71 | (0.39,1.32) | 0.88 | (0.48,1.64) |
| Model 3 | Reference | 1.77 | (0.74,4.21) | 0.59 | (0.30,1.19) | 0.83 | (0.41,1.67) |

HR: hazards ratio; 95%CI: 95% confidence interval; * p<0.05, ** p<0.01, *** p<0.001.

Model 1: unadjusted model; Model 2: adjusted for age and sex; Model 3: adjusted for age, sex marital status, current residence, education level, smoking, drinking, number of chronic conditions, cognitive function, depression and night sleep duration (in the unstratified analyses).
