# Supplementary material for: Association between daytime nap duration and risks of frailty: Findings from the China Health and Retirement Longitudinal Study
Source: Front Public Health. 2023 Jan 27;10:1098609. doi: 10.3389/fpubh.2022.1098609 (PMC9911424; doi:10.3389/fpubh.2022.1098609)
Supplement: Supplementary file 3 [file Table_3.docx]

**Table S3 Comparison of baseline characteristics between participants included and excluded from the study (n= 7 450)**

| Characteristics |  | Included participants (n=5126) | Excluded participants (n=2 324) | p-value |
| --- | --- | --- | --- | --- |
| Age, mean±SD |  | 67.7±6.4 | 69.9±7.9 | <0.001 |
| Gender, n (%) | Male | 2630 (51.3) | 1114 (47.9) | 0.007 |
|  | Female | 2496 (48.7) | 1210 (52.1) |  |
| Marital status, n (%) | Married | 4059 (79.2) | 1708 (73.7) | <0.001 |
|  | Widowed | 961 (18.7) | 548 (23.6) |  |
|  | Others | 106 (2.1) | 63 (2.7) |  |
| Current residence, n (%) | Urban | 1087 (21.2) | 703 (30.4) | <0.001 |
|  | Rural | 4035 (78.8) | 1612 (69.6) |  |
| Education, n (%) | No formal education or illiterate | 1838 (35.9) | 942 (40.8) | <0.001 |
|  | Did not finish elementary school | 1018 (19.9) | 393 (17.0) |  |
|  | Elementary school | 1344 (26.2) | 484 (21.0) |  |
|  | Middle school | 617 (12.0) | 265 (11.5) |  |
|  | High school or above | 308 (6.0) | 224 (9.7) |  |
| Smoking, n (%) | Non-smoker | 2897 (56.5) | 1380 (67.3) | <0.001 |
|  | Ex-smoker | 624 (12.2) | 204 (9.9) |  |
|  | Current smoker | 1605 (31.3) | 467 (22.8) |  |
| Drinking, n (%) | Never | 3535 (69.0) | 1652 (73.1) | 0.002 |
|  | Drink occasionally | 351 (6.8) | 138 (6.1) |  |
|  | Drink frequently | 1240 (24.2) | 471 (20.8) |  |
| Number of chronic conditions, n (%) | 0 | 1387 (27.6) | 603 (27.6) | 0.28 |
|  | 1 | 1596 (31.8) | 656 (30.0) |  |
|  | >1 | 2043 (40.6) | 926 (42.4) |  |
| Cognition score, median (IQR) |  | 10.5 (6.5, 13.5) | 7.0 (2.0, 12.5) | <0.001 |
| Depression, n (%) | No | 3361 (68.8) | 931 (61.6) | <0.001 |
|  | Yes | 1525 (31.2) | 580 (38.4) |  |
| Night sleep duration, n (%) | <6h | 2931 (57.6) | 909 (52.4) | <0.001 |
|  | 6-9h | 1743 (34.3) | 635 (36.6) |  |
|  | ≥9h | 412 (8.1) | 192 (11.1) |  |
| Night Sleep duration, median (IQR) |  | 6.0 (5.0, 8.0) | 6.0 (5.0, 8.0) | 0.44 |

SD, standard deviation; IQR, interquartile range.
